# Chromatin Landscapes of Retroviral and Transposon Integration Profiles

**DOI:** 10.1371/journal.pgen.1004250

**Published:** 2014-04-10

**Authors:** Johann de Jong, Waseem Akhtar, Jitendra Badhai, Alistair G. Rust, Roland Rad, John Hilkens, Anton Berns, Maarten van Lohuizen, Lodewyk F. A. Wessels, Jeroen de Ridder

**Affiliations:** 1 Computational Cancer Biology Group, Division of Molecular Carcinogenesis, The Netherlands Cancer Institute, Amsterdam, The Netherlands; 2 Netherlands Consortium for Systems Biology, Amsterdam, The Netherlands; 3 Division of Molecular Genetics, The Netherlands Cancer Institute, Amsterdam, The Netherlands; 4 Wellcome Trust Sanger Institute, Genome Campus, Hinxton-Cambridge, United Kingdom; 5 Department of Medicine II; Klinikum Rechts der Isar; Technische Universität München, German Cancer Research Center (DKFZ), Heidelberg, & German Cancer Consortium (DKTK), Heidelberg, Germany; 6 Skoltech Center for Stem Cell Research, Skolkovo Institute for Science and Technology, Skolkovo, Odintsovsky, Moscow, Russia; 7 Delft Bioinformatics Lab, Faculty of EEMCS, TU Delft, Delft, The Netherlands; Robert Wood Johnson Medical School, United States of America

## Abstract

The ability of retroviruses and transposons to insert their genetic material into host DNA makes them widely used tools in molecular biology, cancer research and gene therapy. However, these systems have biases that may strongly affect research outcomes. To address this issue, we generated very large datasets consisting of 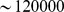 to 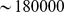 unselected integrations in the mouse genome for the Sleeping Beauty (SB) and piggyBac (PB) transposons, and the Mouse Mammary Tumor Virus (MMTV). We analyzed 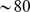 (epi)genomic features to generate bias maps at both local and genome-wide scales. MMTV showed a remarkably uniform distribution of integrations across the genome. More distinct preferences were observed for the two transposons, with PB showing remarkable resemblance to bias profiles of the Murine Leukemia Virus. Furthermore, we present a model where target site selection is directed at multiple scales. At a large scale, target site selection is similar across systems, and defined by domain-oriented features, namely expression of proximal genes, proximity to CpG islands and to genic features, chromatin compaction and replication timing. Notable differences between the systems are mainly observed at smaller scales, and are directed by a diverse range of features. To study the effect of these biases on integration sites occupied under selective pressure, we turned to insertional mutagenesis (IM) screens. In IM screens, putative cancer genes are identified by finding frequently targeted genomic regions, or Common Integration Sites (CISs). Within three recently completed IM screens, we identified 7%–33% putative false positive CISs, which are likely not the result of the oncogenic selection process. Moreover, results indicate that PB, compared to SB, is more suited to tag oncogenes.

## Author Summary

Retroviruses and transposons are widely used in cancer research and gene therapy. However, these systems show integration biases that may strongly affect results. To address this issue, we generated very large datasets consisting of  to  unselected integrations for the Sleeping Beauty and piggyBac transposons, and the Mouse Mammary Tumor Virus (MMTV). We analyzed  (epi)genomic features to generate bias maps at local and genome-wide scales. MMTV showed a remarkably uniform distribution of integrations across the genome, and a striking similarity was observed between piggyBac and the Murine Leukemia Virus. Moreover, we find that target site selection is directed at multiple scales. At larger scales, it is similar across systems, and directed by a set of domain-oriented features, including chromatin compaction, replication timing, and CpG islands. Notable differences between systems are defined at smaller scales by a diverse range of epigenetic features. As a practical application of our findings, we determined that three recent insertional mutagenesis screens - commonly used for cancer gene discovery - contained 7%–33% putative false positive integration hotspots.

## Introduction

DNA integrating elements, such as transposons and retroviruses, are an important tool in many areas of molecular biology, e.g. gene therapy [1], [2], oncogene discovery [3], [4], gene regulation [5], [6], and functional genetics [7], [8]. A current limitation to the use of retroviruses and transposons is that, even without selective pressure, integration loci are not uniformly distributed across the genome. There are significant biases, the molecular determinants of which are still largely unknown. Such biases can pose problems, for example in the discovery of novel cancer genes by insertional mutagenesis (IM), because it can be difficult to distinguish clusters of integrations arising purely through integration bias from those giving a selective growth advantage to the cell. More insight into target site selection would also benefit gene therapy, where adverse integrational activation of oncogenes resulting from treatment with retroviral vectors has been observed [9].

Three of the main integrating elements currently used in the fields mentioned above are the Sleeping Beauty transposon (SB), the piggyBac transposon (PB), and the mouse mammary tumor virus (MMTV). During the last decade, some studies have reported on integration biases in the mouse genome for one or more of these systems. SB does not integrate randomly on a micro-scale, since it is dependent on local DNA deformability, and the presence of a TA dinucleotide at the site of integration [10], [11]. At larger scales integration target site selection was found to be relatively random [12], [13], although (sometimes conflicting) associations have been observed for CpG islands, gene density, and actively transcribed loci [14], [15]. PB integration is TTAA-specific, although slight variations on this target sequence have been observed [16]. PB was found to be biased towards transcriptional units, CpG islands and transcription start sites (TSSs) and actively transcribed loci, and in general marks of open chromatin [14], [17]–[19]. MMTV is the least well-characterized of the three. In mouse and human cell lines, no bias was detected with respect to genes, TSSs and CpG islands, and MMTV was suggested to be the retrovirus least biased in its target site selection [20].

While the studies mentioned above have provided valuable insights into retroviral and transposon target site selection for SB, PB and MMTV in the mouse genome, they do have some limitations with respect to gaining insight into de novo integration target site selection. These limitations can be subdivided into three categories. First, there are limitations regarding the individual integration datasets. For example, some datasets were generated using cells that were enriched with a selectable marker, e.g. [15], [18], [19]. Also, considering only the datasets that were not under selective pressure, the sample sizes were fairly small compared to current standards, mostly in the range of several tens to several hundreds of integrations, e.g. [14]–[16]. Note that having large numbers of integrations is important for gaining sufficient statistical power to detect even relatively weak biases. Second, some limitations complicate the comparison between integration datasets. For example, integration datasets have been compared that differed substantially in the cell lines used, as well as the degree of selection imposed on those cell lines, e.g. [15]. Third, other limitations concern the features used to analyze the integration datasets. For example, integration datasets have been compared to features in non-matching cell types [15], while for example Murine Leukemia Virus target site selection within the human genome has been suggested [21] and shown [22] to have a cell type dependent component. Interesting to note here is that for a resurrected human endogenous retrovirus, no cell type specific integration into the human genome could be detected [23]. Also, many studies focused only on a limited number of features, e.g. genomic features [17], [20], or genomic features and DNase I hypersensitivity [15]. Moreover, the features, such as ChIP-seq profiles, were not necessarily preprocessed in similar ways, for example in terms of sequence alignment, e.g. [18]. This complicates the comparison of features across different systems.

To address these questions, we generated large datasets of SB and PB integrations in mouse embryonic stem cells (mESC). In order to directly compare the two transposons, they were mobilized from the same construct containing inverted repeats (IRs) for both PB and SB. This eliminates any other possible cause than the IR (or the transposon-specific transposase) for the observed differences between the two systems. In addition, we generated a large dataset of MMTV integrations in normal murine mammary gland epithelial (NMuMG) cells. All three datasets were generated under minimal selective pressure, and are henceforward referred to as unselected integration profiles. They are considerably larger than previously published datasets of unselected integrations, 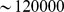, 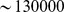, and 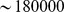 integrations respectively.

We associated the three integration profiles with a large number of genomic and epigenomic features. In particular, for SB and PB, the recent explosive growth in publicly available ChIP-seq datasets [24] enabled us to analyze a large number of epigenomic features (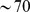), all in mESCs. To allow for a better comparison between these datasets, they were preprocessed from the raw sequence reads in exactly the same way.

Additionally, the impact of selective pressure on an unselected integration profile, which is important in using IM for cancer gene discovery, has never been addressed extensively. Previous work can be classified as either knowledge-based or data-driven. The knowledge-based approaches use modeling of previously described integration biases to avoid CIS calls that can be explained by these biases, such as SB TA sequence specificity [25], or 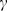-retroviral TSS specificity and lentiviral gene specificity [26]. Alternatively, by assuming that a genic region harboring a true positive CIS should contain significantly more integrations than its flanking genes, three out of nine CISs from gene therapeutic clinical trials were labeled false positive [27]. These knowledge-based approaches are necessarily limited in their modeling of integration bias, for example in the number of features that are considered. Conversely, data-driven approaches treat integration bias as a black box, and compare integration datasets that were under substantial selective pressure to integration datasets that lacked this pressure. Using this approach, one study suggested a 47% false positive rate for a MuLV tumor screen [28], and another observed 6 control CISs from SB integrations present in mouse tail DNA, where 79 CISs could be found in the corresponding SB tumor screen [29]. To analyze the impact of selective pressure on integration bias profiles, we take the data-driven approach and compare our three unselected datasets with CIS integration profiles from three previously published tumor screens [3], [30]–[32].

Taken together, this allows us to present the most extensive analysis of SB, PB and MMTV target site selection to date, the results of which include previously undetected biases and differences between selected and unselected integration profiles. Another focal point of the analysis is the influence of scale. By analyzing differences between small-scale (within 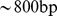 from integrations) and large-scale (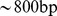 or further from integrations) associations of genomic and epigenomic features with the proximity of integrations, we reveal a hierarchical organization in integration bias. On a global scale, target sites of different systems are selected in similar ways, whereas differences mainly exist in fine-tuning on a local scale.

## Results

The integration datasets used in this study are described in Table 1. For each tumor screen, the numbers of singleton integrations and CIS integrations is given. Here, a CIS is a genomic region with more integrations across tumors than expected by chance (see Material and Methods section and [3], [33]). An integration falling (not falling) within a CIS is referred to as a CIS (singleton) integration.

**Table 1:** Integration datasets used in this paper.

System	Cell type	Size	Selected	#CIS insertions	#Singleton insertions	Reference
MuLV	B/T-cell tumors	20312	Yes	8707	11605	[3], [37]
SB	Mouse ES cells	131594	No	NA	NA	This work
SB	B/T-cell tumors	58266	Yes	1945	56321	[30]
PB	Mouse ES cells	122667	No	NA	NA	This work
PB	Haematopoietic tumors	5590	Yes	306	5284	[32]
MMTV	Mouse mammary cells	180469	No	NA	NA	This work
MMTV	Mouse mammary tumors	34753	Yes	2834	31919	[31]

The features for which integration bias was studied are summarized in Table 2. Statistical procedures used for each figure are listed in Table S1.

**Table 2:** Genome and chromatin profiling data used in this paper.

Description	Technique	Cell type	Reference
Gene expression mESC	RNA-seq	mESC	This work
Gene expression NMuMG	Microarray	NMuMG	[67]
Replication timing	ChIP-chip	mESC	[65]
H3K9me2	ChIP-chip	mESC	[64]
LaminB1	DamID	mESC	[66]
Hi-C	Hi-C	mESC	[39]
mC; hmC	Bisulfite sequencing; hMeDIP	mESC	[61]
CpG island proximity; gene proximity	NA	NA	[67]
Dnase I hypersensitivity	ChIP-seq	mESC	[69]
H3K4me3; H4K20me3	ChIP-seq	mESC	[49]
H3K9me3	ChIP-seq	mESC	[70]
H3K27me3; H3K36me3; H2AZ	ChIP-seq	mESC	[71]
Atrx	ChIP-seq	mESC	[72]
BrgJ1	ChIP-seq	mESC	[73]
cMyc; E2f1; Esrrb; Klf4; nMyc; Oct4; Smad1; Sox2; Stat3; Suz12; Tcfcp2|1; Zfx	ChIP-seq	mESC	[74]
H3K79me2; Nanog; Tcf3	ChIP-seq	mESC	[75]
Ezh2	ChIP-seq	mESC	[76]
SetDB1	ChIP-seq	mESC	[77]
Jarid2; Mtf2	ChIP-seq	mESC	[78]
Tbx3	ChIP-seq	mESC	[79]
Ctr9; Pol2-Ser2P; Pol2-Ser5P	ChIP-seq	mESC	[80]
Luzp1	ChIP-seq	mESC	[81]
Chd7	ChIP-seq	mESC	[82]
Med1; Med12; Smc1; Smc3	ChIP-seq	mESC	[48]
H3K27ac; H3K4me1	ChIP-seq	mESC	[51]
Yy1	ChIP-seq	mESC	[83]
CTCF; p300	ChIP-seq	mESC	[84]
H3K9ac	ChIP-seq	mESC	[85]
Taf3	ChIP-seq	mESC	[86]
Jaridb1	ChIP-seq	mESC	[87]
Smad2/3; Smad3	ChIP-seq	mESC	[88]
Kap1	ChIP-seq	mESC	[89]
H3K4me2	ChIP-seq	mESC	[90]
macroH2A1	ChIP-seq	mESC	[91]
p53; p53S18P	ChIP-seq	mESC	[92]
Cbx7; Ring1b	ChIP-seq	mESC	[93]
CoREST; Hdac1; Hdac2; Lsd1; Mi-2b	ChIP-seq	mESC	[94]
Dpy30	ChIP-seq	mESC	[95]
Mbd3	ChIP-seq	mESC	[96]
Mcaf1	ChIP-seq	mESC	[97]
Pol2-Ser7P	ChIP-seq	mESC	[98]
Tbp	ChIP-seq	mESC	[99]
Ell	ChIP-seq	mESC	[100]

### SB, PB, and MMTV exhibit unique sequence and gene specificity

We started our analysis by studying the sequence specificity at integration sites of the three systems. As expected, PB and SB mostly, but not exclusively, integrate at TTAA and TA sites respectively, accounting for 93% (PB) and 94% (SB) of the integrations (Figure S1). The remaining integrations show sequences that are relatively similar to these motifs and often differ by only one nucleotide (Figure S1). MMTV has little integration site sequence specificity. Focusing instead on integration-flanking sequences (50 bp on either side), de novo motif discovery using the HOMER software [34] revealed no enrichment of non-trivial motifs (i.e. not TTAA for PB, and not TA for SB), except for two motifs in the case of MMTV (binding TFAP2A and Tcfap2e respectively; Figure S2).

**Figure 1:**
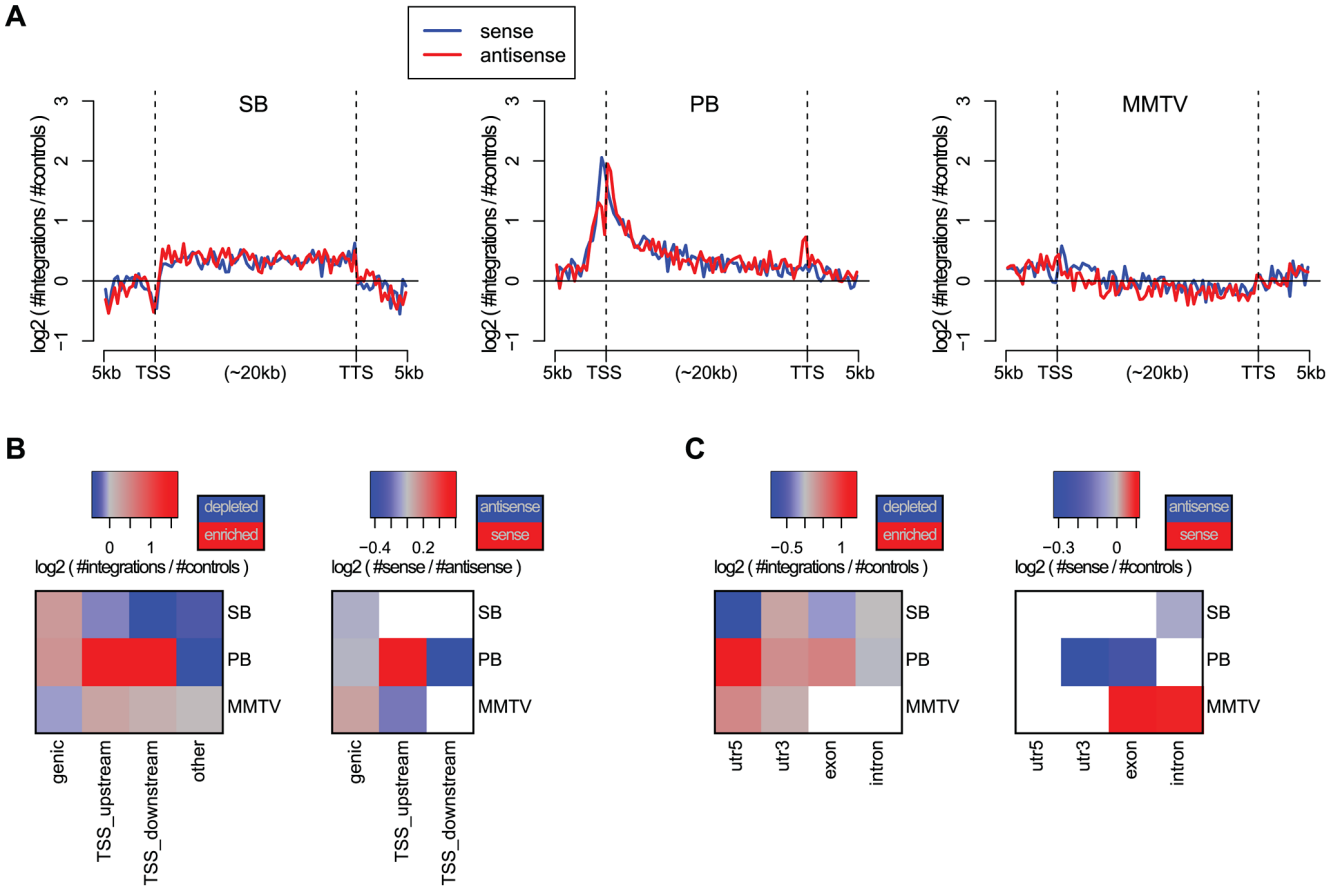
Biases with respect to genes and transcripts. A) Gene alignment plots showing the distribution of integrations across genes from 5 kb upstream to the transcription start site (TSS), transcription termination site (TTS), and 5 kb downstream. The red line depicts the integrations with sense orientation relative to the gene, blue depicts antisense. B) Biases with respect to genes for the unselected integration datasets. We distinguish between integrations within genes (genic), within 1 kb upstream of the TSS (TSS_upstream), within 1 kb downstream of the TSS (TSS_downstream), and other integrations (other). On the left, the color scale blue-gray-red represents increasing numbers of integrations, relative to expected. On the right, the color scale blue-gray-red represents the integration orientation bias relative to genes, from antisense to sense. Associations that are not significant (binomial test; FDR-corrected 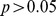) are white. C) Biases with respect to transcripts, distinguishing between integrations in 5′UTRs, 3′UTRs, exons and introns.

The bias of an integrating element with respect to genes is of particular interest in IM and gene therapy. In IM screens, more integrations near genes are desired whereas in gene therapy integrations proximal to genes pose a potential threat to the patient, since such integrations may give rise to cancer. We, therefore, compared the integration density of the three systems in and around genes, by aligning all genes (Figure 1A). PB shows a strong bias for TSSs. The PB bias profile with respect to genes is remarkably similar to that of MuLV, see e.g. [35], [36], as well as Figure S3 which shows singleton MuLV profiles, based on a previously published tumor screen [3], [37]. While PB has a strong bias for TSSs, SB is enriched uniformly along the body of genes. MMTV shows the weakest bias, although it does slightly prefer TSSs, and has a mild bias against gene bodies.

The significance of the observations made above was assessed using the binomial test, and visualized at a 5% FDR threshold in Figures 1B and 1C, for different gene and transcript related regions. Refer to Table S2 for the raw *p*-values associated with these statistical tests. It confirms the strong bias of PB for TSSs, and indicates that PB prefers integrating with its transcription unit oriented towards the TSS. When landing within genes, MMTV prefers a sense orientation relative to the host gene, and SB and PB an antisense orientation. In general, MMTV shows the least biased profile, with weak but significant biases for TSSs, and against genic regions. PB integrations are mainly enriched in the 5′UTR, weakly enriched in exons and the 3′UTR, and biased against introns. This pattern is highly similar to that observed for singleton MuLV integrations (Figure S4). Regarding orientation biases within transcripts, MMTV shows sense orientation biases for exons and introns, whereas the two transposons show only antisense orientation biases, SB in introns, and PB in 3′UTRs and exons.

### Integration profiles of SB and PB are shaped by endogenous gene expression

**Figure 2:**
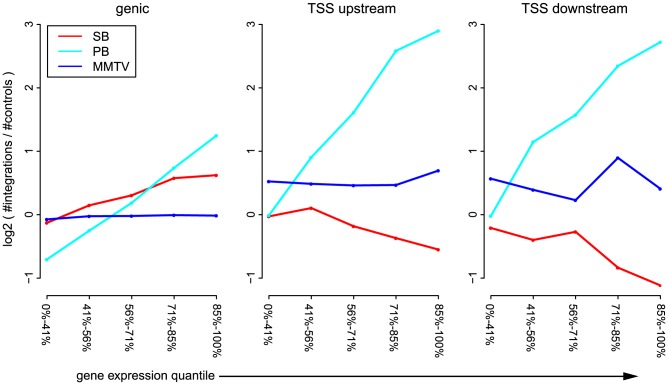
Influence of gene expression on integration bias. For each of the systems SB, PB, and MMTV, the unselected integrations are divided into genic integrations, integrations occurring within 1(TSS_upstream), and integrations occurring within 1 kb downstream of the TSS (TSS_downstream). Genes are divided into 5 groups, based on expression level. The sizes of these groups are indicated on the 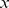-axis. For each pair of gene expression level and system, the number of observed integrations is counted, and compared to the number of expected integrations.

Next, we analyzed the influence of expression status of endogenous genes on integration bias. This revealed interesting differences between the unselected integration profiles of the three systems (Figure 2), significance of which was assessed by the Cochran-Armitage trend test, unless mentioned otherwise. Across genes and TSSs, PB is strongly influenced by the a priori gene expression levels (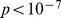 for genic, TSS_upstream and TSS_downstream). For SB this same positive trend is only apparent for intragenic integrations (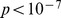), whereas around TSSs, the numbers of integrations decrease with increasing gene expression (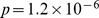 and 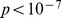 for TSS_upstream and TSS_downstream respectively). Within weakly expressed genes, there is a depletion of PB integrations (binomial test; 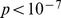). MMTV target site selection is largely independent from the expression levels of endogenous genes (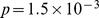, 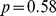 and 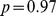 for genic, TSS_upstream and TSS_upstream respectively). Although it is evident that there are more MMTV integrations in TSS regions than within genes (Figures 1 and 2), this preference is clearly independent from gene expression.

### Topological domain interfaces are hotspots of integration

**Figure 3:**
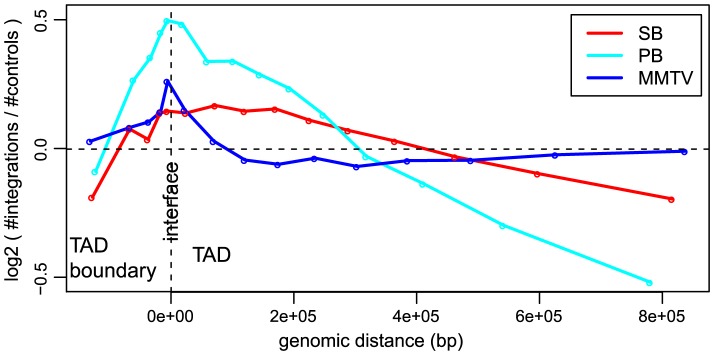
Unselected integration profiles with respect to TAD - TAD boundary interface. The 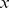-axis represents genomic distance from the interface. The 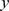-axis represents the log2 ratio of observed number of integrations versus the expected number of integrations.

In addition to gene structure, the integration site selection of an integrating element can also be influenced by other features such as organization of the genome, state of chromatin compaction and transcription factor binding events, as well as by epigenetic modifications. Considering that an important barrier for integration of viral or transposon DNA into host DNA can be how tightly the DNA is packed in chromatin, we looked at the influence of the a priori chromatin organization on the unselected integration profiles. Hi-C [38] is a technique for studying chromatin compaction and organization by determining interaction frequencies between different genomic loci on a genome-wide scale. Analysis of Hi-C data has suggested that the genome is organized into chromatin modules, called topologically associated domains (TADs), which are stable across different cell types [39]. TADs are separated by less organized (showing fewer 3D interactions) regions called TAD boundaries. It is conceivable that chromatin is relatively less compact in and near TAD boundaries as compared to TADs. We asked if this 3D organization of the genome has any influence on the integration bias of systems. In general, we found that more integrations are close to the interface between TADs and their boundaries (Figure 3), i.e. all systems have a preference for inserting at the border of TADs, which are tightly organized, but not necessarily in the less organized chromatin of boundary regions. It is interesting to note that for MMTV, which is generally the least biased system, the bias for the TAD - TAD boundary interface is stronger than that of SB (Cochran-Mantel-Haenszel test in a window of 10 kb on either side of the interface; 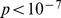).

### Transposons show highly divergent behavior in integrative (epi)genomic context

In the previous two sections, we demonstrated that there are strong biases of the unselected integration profiles with respect to genes, transcripts, gene expression, and genome organization. However, these features themselves have strong spatial ties with other features, such as histone marks and transcription factor binding. Therefore, we asked the following two questions. First, how do these features associate with integration proximity? Second, do they provide extra information with respect to integration bias, in addition to what gene proximity and gene expression provide? The features we analyzed are listed in Table 2. To maximize comparability between the features, the ChIP-seq datasets were preprocessed from the raw sequencing reads in exactly the same way. Since these features are not available in NMuMG cells, which were used for generating the MMTV integrations, we restricted all analyses based on these data to SB and PB.

First, we analyzed the orientation biases with respect to these features (Figures S5 and S6). This showed that the two transposons preferably integrate with the transcription cassette cloned in them oriented towards regions of high feature signal. Although for individual marks this bias is not very substantial, it is highly consistent across different marks, especially for SB. It is important to note that an orientation bias of these systems relative to genes cannot explain this bias for SB, and only partly for PB (Figures S5 and S6).

**Figure 4:**
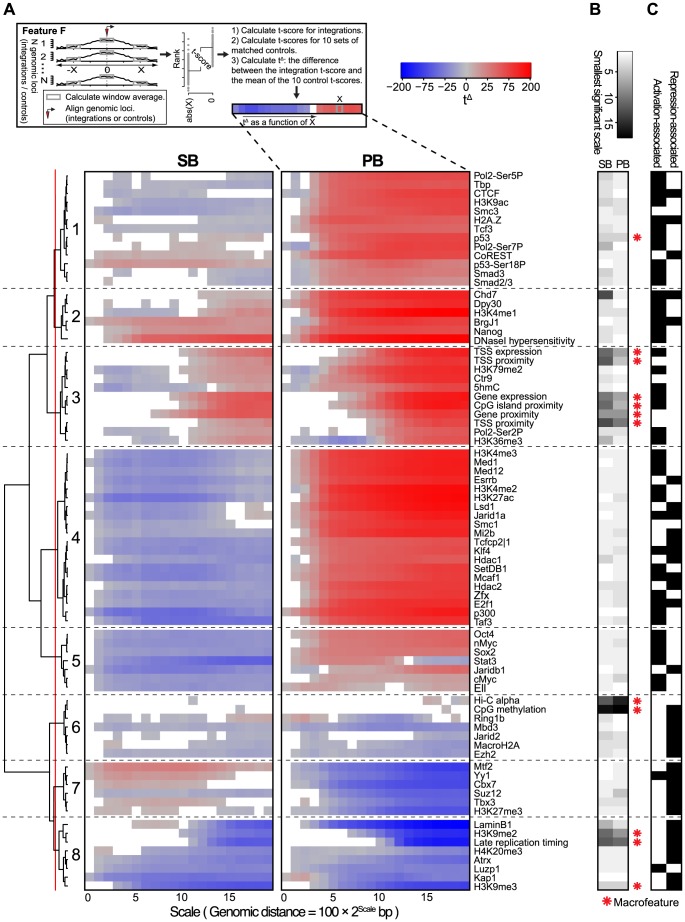
Scale-based analysis of integration bias. A) Association of the unselected integration profiles with various genome-wide features across different scales. Measure of association is a normalized *t*-score (see Material and Methods), computed on rank-normalized feature values, visualized on a blue-gray-red scale from negative to positive *t*-scores. Associations that are not significant (FDR-corrected 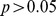) are white. A positive (negative) *t*-score for a certain scale 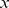 and feature 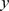 means that for that particular feature, the mean values in a 200 bp window around the integrations are on average higher (lower) than the mean values in a 200 bp window around the points at a distance of 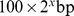 upstream and downstream from the integration (see Material and Methods). The dendrogram shows a hierarchical clustering of the profiles using the euclidean distance measure and ward linkage. B) The rank-transformed smallest scale at which significance is achieved, with a scale going from white (small scale) to black (large scale). A feature is called a ‘macrofeature’ if its smallest significant scale is larger than the mean rank-normalized smallest significant scale across features, in both systems. C) Features associated with transcriptional repression and/or activation, based on published literature.

Using a limited number of mostly genomic features, it has been observed before that associations of integration occurrence with these features depend on the scale chosen for the analysis [15]. Therefore, we analyzed our genomic and epigenomic features across different scales, by comparing feature scores at the site of integration with feature scores at increasing distances (scales) from the integration site. Features were then clustered based on their association profiles across scales (Figure 4A). Resulting associations can be positive, i.e. higher feature scores at integration sites compared to their neighborhood, or negative, i.e. lower feature scores at integration sites compared to their neighborhood.

A clustering of the association profiles results in four groups of features generally associated with activation (Clusters 1, 2, 3 and 5), and three groups associated with repression (Clusters 6, 7 and 8). The remaining Cluster 5 is more mixed (Figure 4C).

Another characterization of the resulting clusters is into groups of features for which the behavior is either fairly similar (Clusters 2, 3, 6 and 8), or groups for which SB and PB behave very differently (Clusters 1, 4, 5, and 7). Especially striking when observing the differences is that PB is positively associated (Clusters 1, 4 and 5) with far more features than SB (Cluster 7). Since for both PB and SB, association with the many gene-related features in Cluster 3 is mostly positive, this indicates that SB does prefer gene-rich regions and active genes over heterochromatin, but in these regions, compared to PB, generally avoids regulatory units such as histone modifications and transcription factor bound regions. Interestingly, the single cluster positively associating with SB but negatively with PB (Cluster 7) contains mostly repressive features. Combined, these observations suggest that PB is much more biased to active chromatin than SB, whereas SB is also partly biased towards more repressed chromatin.

The scale-based approach reveals that the sign of association can change across different scales. For example, SB shows negative association with some of the features in Cluster 3 on a small scale, but a positive association on larger scales. This implies that SB has a bias for larger scale domains containing these features such as Ctr9, H3K79me2 and 5hmC, but within these domains integration sites will generally avoid overlap with these marks.

Conversely, association changing from positive to negative for increasing scales is seen for example for PB and Stat3 in Cluster 5. This indicates that PB prefers domains relatively devoid of these features. However, given a PB integration in such a domain, it will be mildly biased towards Stat3.

The above observations suggest a hierarchy in target site selection, which is further illustrated by the fact that some features, for both SB and PB, are consistently non-significant at smaller scales (Figures 4A and 4B). For example, at smaller distances from integrations, associations with features such as gene proximity and expression, CpG island proximity, replication timing and H3K9me2 are not significant for both SB and PB. They are however consistently significant on larger scales. On the other hand, most transcription factors and other histone marks show strong associations already at small scales. Henceforward, features that are significant only at larger scales will be referred to as ‘macrofeatures’, as opposed to ‘microfeatures’, which are significant already at smaller scales (refer to Table S3 for a list of macrofeatures and microfeatures). For a selected set of macrofeatures that were available for NMuMG cells, we performed a similar analysis showing that these features also behave as macrofeatures in MMTV, an unrelated system (Figure S7).

### Integration site selection is directed at multiple levels

Biases of unselected integration profiles with respect to the macrofeatures are similar across the systems and scales. This suggests that on a large scale, integration bias is regulated in similar ways for both systems, and that this large scale bias is mainly determined by the macrofeatures. However, within a distance of 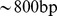, macrofeatures provide no information with regard to integration locus, contrary to the microfeatures. This indicates that microfeatures may in fact be determinants of integration bias at a higher resolution, which prompted us to ask the following question: Are macrofeatures needed at all to explain integration proximity, or are microfeatures sufficient for this purpose?

To address this question, we needed to take into account that the features in Figure 4 show a high degree of multicollinearity. Multicollinearity implies that a strong association between a certain feature 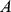 and integration proximity may potentially be explained by the association of 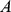 with another feature 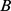 that also strongly associates with integration proximity, i.e. integration proximity may be conditionally independent from 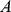, given 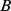. Then, rephrasing the question above, for each system we wanted to identify a set of features such that integration proximity is conditionally independent from all other features, given this set of features.

BANJO [40] is designed to identify such conditional independencies in the form of Bayesian networks [41], and thus allowed us to determine for each feature its importance for integration proximity. For this, we used two measures derived from the Bayesian networks. The first measure (‘log10% bootstraps’; see Material and Methods) represents the confidence that a feature is truly relevant for integration proximity. The second measure (‘log10 mean CMI’, or conditional mutual information; see Material and Methods) represents the strength of association between integration proximity and a feature.

**Figure 5:**
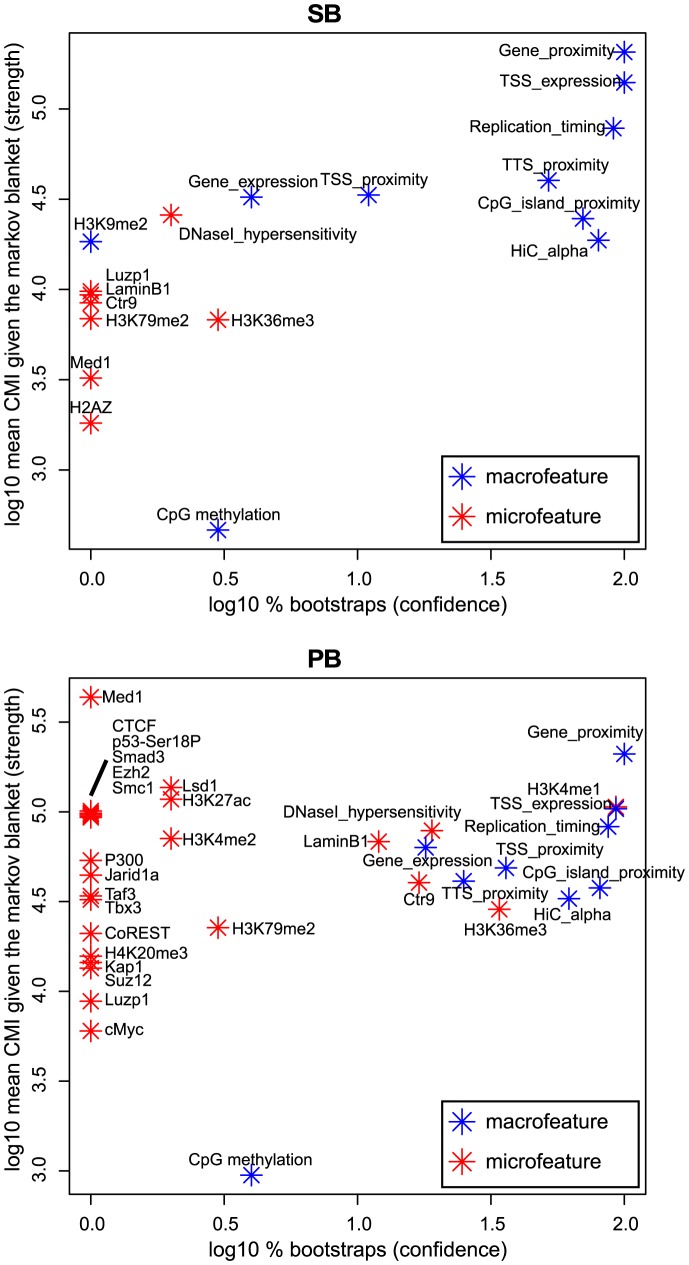
Bootstrapped Markov blanket discovery. Bayesian network inference (BNI) is performed on 400 bootstraps of size 20000. The 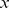-axis represents the fraction of bootstraps that a feature occurs in the Markov blanket of integration proximity in a resulting Bayesian network, i.e. the confidence we have in an edge. The 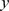-axis represents the mean conditional mutual information (CMI) of integration proximity with a feature across all Markov blankets in which this feature occurs, i.e. the strength of an edge. Note that features that do not occur in the Markov blanket of any bootstrap, i.e. are never considered relevant for integration proximity by the BNI approach, are not shown in this figure.

Interestingly, the results show that seven macrofeatures are consistently of great importance, i.e. of high-confidence and strongly associating, in both systems (Figure 5). These features are gene/TSS/TTS proximity, TSS expression (the expression of the gene with the nearest TSS), replication timing, CpG island proximity, and Hi-C alpha (a measure of chromatin compaction; see Material and Methods section). This shows that in addition to microfeatures for explaining local differences between systems (Figure 4), macrofeatures are needed to explain integration bias in each of these systems on large scales (Figure 5). Furthermore, it indicates that on a large scale, biases of the two systems are similar, and that differences between the systems are mainly found in the microfeatures.

### Integration bias is a potential cause of spurious common integration sites

Insertional mutagenesis (IM) using retroviruses and transposons is an important tool in the discovery of new putative cancer genes. These elements mutate the genome by inserting into the host DNA. Mutations providing cells with a proliferative and/or survival advantage can cause tumors. Because the integration loci can be retrieved using sequencing, these mutations can act as cancer gene tags, allowing discovery of novel cancer genes, e.g. [3], [4], [29], [37], [42]–[44]. However, integration biases can pose problems because they can be difficult to distinguish from the accumulation of integrations in cells that are under selective pressure to retain these integrations. Therefore, we compared the unselected integration profiles with CIS integration profiles [3], [30]–[32].

**Figure 6:**
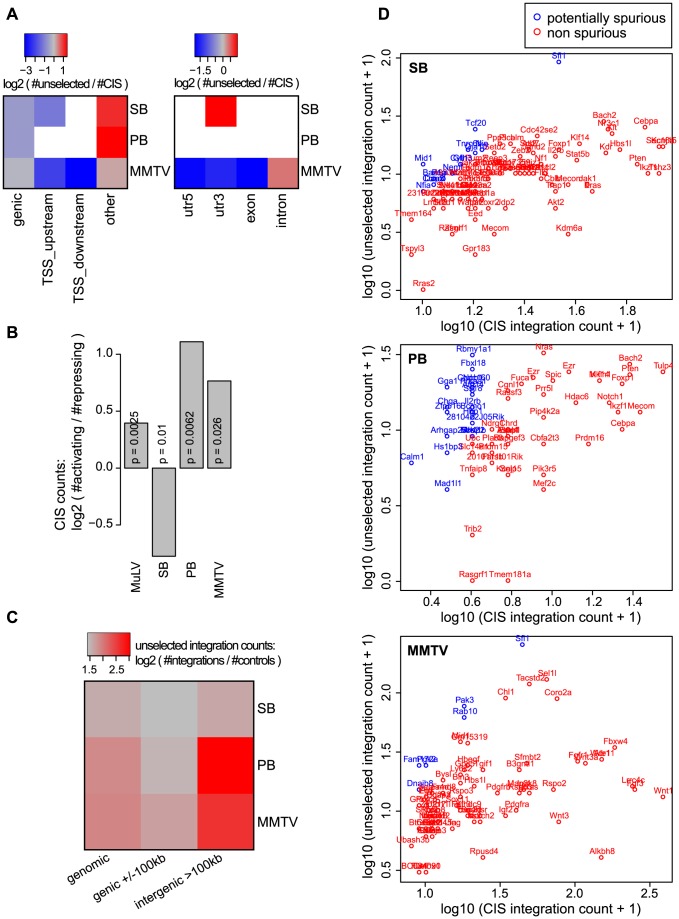
Unselected integration profiles and CIS designation. A) The bias of unselected integrations relative to CIS integrations, on a scale from blue (more CIS integrations) to red (more unselected integrations). B) log2 ratio of activating CISs and repressing CISs. A CIS is activating if it is not within a gene, or within a gene and 90% homogeneous with regard to orientation relative to that gene. Otherwise it is repressive. C) Bias of unselected integrations for CIS regions in a (i) genome-wide background, (ii) genic background (+/−100 kb), and (iii) intergenic background (whole genome except genes +/−100 kb), as measured by the log2 ratio of observed (unselected integrations) and expected (matched controls). D) CIS integration counts vs. unselected integration counts. CISs are annotated with the nearest TSS. Note that a single gene can be associated with multiple CISs. Spurious CISs were determined by a one-sided binomial test to determine if the CIS contained more CIS integrations than unselected integrations (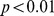, FDR-corrected).

Generally, the orientation biases of CIS integrations for genes and transcripts are much stronger than those of the unselected integrations (Figure S4). This indicates that in tumors, the orientation bias is mainly the result of selective pressure. For all systems and especially for PB and SB, there are significantly more unselected integrations than CIS integrations in regions other than genes and TSSs (Figure 6A; visualized at a 5% FDR threshold based on the binomial test. Refer to Table S2 for the raw *p*-values). Additionally, biases of unselected integrations for intergenic CIS regions (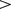
100 kb from genes) are relatively strong, compared to the biases for genic CIS regions (+/−100 kb) (Figure 6C; visualized at a 5% FDR threshold based on the binomial test. Refer to Table S2 for the raw *p*-values). Combined, these observations show that in regions far from genes, unselected integration profiles correlate relatively strongly with CIS profiles, compared to regions close to genes. Therefore, to avoid calling spurious CISs in IM screens, higher statistical stringency should be required for CISs found far from genes.

Combined, these observations suggest that CISs found far away from genes are more likely to be spurious, i.e. arise from the integration bias of the system. Conversely, the observations suggest that true CISs, i.e. CISs arising from selective pressure, are more often found in the vicinity of genes than would be expected based on an unselected integration profile.

To identify potentially spurious CISs, we tested for all CISs if the corresponding CIS regions contained significantly more CIS integrations than unselected integrations. This revealed a number of potentially spurious CISs, 13%, 33% and 7.4% of all CISs for SB, PB and MMTV respectively (Figure 6D, Tables S4, S5 and S6). For MMTV, it could be confirmed that potentially spurious CISs tended to be relatively far away from genes (One-sided Mann-Whitney U test; 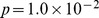). For SB and PB, when ranking CISs according to increasing *p*-value, the potentially spurious CISs consisted mainly of lower ranking CISs (Mann-Whitney U test; 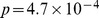 and 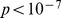 for SB and PB respectively).

Next, we asked whether integration bias has an influence on the types of CISs that are found in screens. For this purpose, we separated CISs into activating and repressing CISs, based on orientation homogeneity and occurrence within or outside genes. We observed more activating CISs for PB than for SB (Figure 6B). Considering that the constructs used for the SB and PB tumor screens are similar [32], [45], this indicates that for use in IM screens, PB is more efficient at finding oncogenes, whereas SB would find more tumor suppressor genes.

## Discussion

In this study, we have analyzed the integration biases of unselected integrations of a retrovirus and two transposons. For generating these sets of integrations, cells were grown in culture for three to four weeks. This implies that a few of our integration loci could potentially have been selected for. However, non-acute retroviruses, such as MMTV, induce tumors only very slowly (months to years) due to the absence of oncogenes in their genome [46]. Similarly, our transposon constructs can be described as non-acute in the sense that they do not carry oncogenes. Moreover, they do not contain any gene-trap or enhancer-trap elements, limiting the potential of disrupting endogenous gene expression. Hence, three or four weeks of cell culturing is a very short time frame compared to the latency to integration-induced tumor formation, and the influence of selected integration loci will be minimal at best. This is also supported by the observations that 1) we find a large number of unique integration sites, whereas in the case of substantial selection a relatively small number of (selected) integrations would be expected, and 2) while both PB and SB were mobilized from the same construct, we do obtain completely different insertion profiles for each transposon.

The main differences between the three systems regarding integration bias are summarized in Table 3. Generally, MMTV was observed to be the system least biased in its integration profile. Although many associations were found to be significant, they were generally not very substantial. In this context, it is surprising that only a small set of oncogenes has been tagged with this virus in IM screens [31], [47]. This could be due to activation of a limited set of promoters by the MMTV enhancer. Alternatively, certain unknown aspects of murine mammary tissue biology might allow only a limited number of tumorigenic mechanisms. In any case, our data rules out integration bias as a reason for the limited potential of IM by MMTV.

**Table 3:** Summary of main observations.

	SB	PB	MMTV
**Sequence**	TA	TTAA	Very weak bias
**Genes**	Whole gene; bias against TSS	TSS; whole gene	TSS; weakly intergenic; bias against gene body
**Transcripts**	3′UTR; bias against 5′UTR/exon	Exon; 5′/3′UTR	5′/3′UTR
**Orientation**	Antisense within genes (introns)	Towards TSS	Sense within genes; upstream away from TSS
**CIS targets**	Tumor suppressor genes	Oncogenes	Oncogenes
**spurious CISs**	Lower ranking CISs	Lower ranking CISs	Farther away from genes
**gene expression**	Intermediate bias	Strong bias	Weak bias
**TAD interface**	Weak bias	Strong bias	Intermediate bias
**Orientation w.r.t. chromatin marks**	Towards chromatin marks (weak but consistent)	Towards chromatin marks (weak but consistent)	NA
**macrofeatures**	Relatively strong biases, consistent across systems: TSS expression, replication timing, Hi-C alpha, CpG island/TSS/TTS/gene proximity	Relatively strong biases, consistent across systems: TSS expression, replication timing, Hi-C alpha, CpG island/TSS/TTS/gene proximity	NA
**microfeatures**	Relatively many associations are negative	Strong positive associations with many features	NA

Recent availability of data describing the three-dimensional architecture of the genome [39] has allowed us to identify TAD - TAD boundary interfaces as hotspots of integration. Of the three systems, PB is most strongly affected, from a strong enrichment at TAD interfaces to a strong depletion towards the inner regions of TADs. While MMTV is largely indifferent regarding these inner TAD regions, its bias for TAD - TAD boundary interfaces is relatively strong. Although the TADs were defined in a different cell type (Tables 1 and 2), they have been shown to be stable across different cell types [39]. Altogether, our data show that integration target site selection is strongly associated with the topological organization of the genome.

The two transposons were found to have very different integration profiles. Generally, it is unknown to what extent certain sequences cloned into an IM construct affect the integration bias of that construct, which complicates the interpretation of differences observed between systems. However, the construct that was used in this study contained both the SB and PB IRs. Therefore, any difference between the two profiles can only be explained by the IR or the transposon-specific transposase, indicating that the IR and transposase are major defining elements of integration bias.

**Figure 7:**
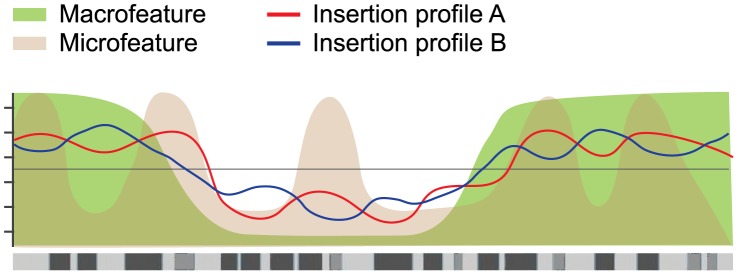
Hierarchical model of integration target site selection. On a large scale, target site selection is directed by macrofeatures for all three systems in similar ways. Differences between the systems are determined by microfeatures.

Although the SB and PB profiles are very different, they were shown to share a bias for activating macrofeatures and a bias against repressive macrofeatures. Analysis of a subset of macrofeatures for MMTV suggested that these may also operate as macrofeatures in a wider range of systems. Differences between SB and PB were mostly seen for the microfeatures. Together, these observations support a model where integration sites are selected at two levels (Figure 7). On larger scales, both systems target the same type of domains, determined by the macrofeatures. In particular, gene/TSS/TTS proximity, TSS expression, replication timing, CpG island proximity, and Hi-C alpha were found consistently indispensable for integration site selection in both systems (Figure 5). Once these domains have been selected, fine tuning of integration site selection is dependent on different microfeatures for each system. These microfeatures appear to be indispensable for integration site selection as well (Figure 4).

For the current study, the mESC model system was selected because it is the most thoroughly studied model system, with the broadest availability of (epi)genomic datasets. While limited cell type specificities have been demonstrated for retroviral integration profiles [22], earlier studies have used epigenomic features in non-matching cell types to analyze retroviral integration profiles [15], [23], noting that differences due to experimental error are generally greater than differences due to cell type [23]. We do not expect our transposon integration profiles to be highly cell type specific. This is supported by a supplementary analysis comparing the SB and PB integration profiles to a selected set of epigenomic features available for both mESC and mouse embryonic fibroblasts (mEF) [48]–[52], which shows that the mEF associations are highly similar to the mESC associations (Figure S8). This strong similarity suggests that cell type specificities are relatively weak. Nevertheless, it is interesting to note that the mEF associations are consistently slightly weaker than the mESC associations, indicating that cell type specificities, while weak, do exist.

In large scale IM screens, identification of overwhelming numbers of CISs is a serious impediment in distinguishing true CISs from spurious ones. In such screens, a true CIS arises through tumorigenic selection, whereas a spurious CIS is defined by the a priori integration bias of the IM system that was used. Our large datasets of unselected integrations can be used as a valuable resource for prioritizing candidate cancer genes emerging from IM screens. As a proof of principle, we showed that a substantial number of CIS regions in three recent IM screens do not contain more integrations than would be expected based on the unselected integration profiles. Although the cell types between PB and SB tumor screens and their corresponding unselected profiles are different, this nevertheless indicates that these CISs can potentially be explained by an a priori integration bias, and therefore likely represent passenger mutations.

In conclusion, the large numbers of integrations for three of the main systems used in IM, unselected integrations from cell lines and selected integrations from tumor screens, as well as the wide range of publicly available datasets, has enabled us to assess integration bias at unprecedented resolution, and assess its relation to CIS designation.

## Materials and Methods

### Data generation

All integration data generated in this study are made available on http://mutapedia.nki.nl.

#### PB and SB integration site data

mES cells EBRTcH3 expressing the tetracycline-controlled transactivator (tTA) from the endogenous ROSA26 promoter [53] were cultured in 60% BRL cell-conditioned medium in the presence of leukemia inhibitory factor, MEK inhibitor PD0325901 and GSK-3 inhibitor CHIR99021 [54]. pPB-SB-CMV-GFP was constructed by cloning GFP CDS in PB-MSCV [55] at Nru-I and BstXI sites. 4 hr before transfection, 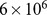 EBRTcH3 cells were seeded on a 10 cm dish. The cells were transfected with 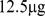 of pPB-SB-CMV-GFP and either 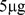 of mPB transposase plasmid [56] or 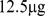 of SB100X transposase plasmid [57] using Lipofectamine 2000 (Invitrogen). Mock transfected and non-transfected controls were included. After 48 hr, 60000–80000 cells were isolated and further propagated (Figure S9). After three weeks of culturing post-transfection the genomic DNA was isolated using Qiagen DNeasy Blood & Tissue kit. 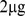 of genomic DNA was digested with 20 units of Dpn-II (New England Biolabs) at 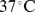 for overnight in a 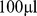 reaction. 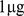 of purified digested DNA was ligated with 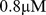 of splinkerette adapter using 10 units of T4 DNA ligase (Roche Applied Science) in a 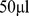 reaction. The splinkerette adapter was prepared by annealing equimolar amounts (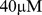 each) of Universal US (GTTCCCATGGTACTACTCATATAATACGACTCACTATAGG) and Sau-3A-1 LS (GATCCCTATAGTGAGTCGTATTATAATTTTTTTTTCAAAAAAA) oligos. The ligation reactions were amplified in two (SB) or three (PB) rounds of PCR to generate libraries for high throughput sequencing (for details see Table S7).

Sequencing was done on an Illumina HiSeq 2000 instrument to obtain single 100 bp reads. The reads contained ends of IRs and the neighboring genomic DNA. The genomic DNA sequences were extracted from sequencing reads, and aligned against mouse genome assembly mm9 using Bowtie 2 [58] to determine the sites and orientation of integrations, using parameter ‘very-sensitive-local’. Only those positions which were represented by five or more reads in the data, were retained and used for subsequent analyses.

#### MMTV integration site data

Mm5MT (MMTV producing cells) and NM-Pbabe/2 (NMuMG cells harboring PuroR transgene) were cultured in DMEM/F-12+GlutaMAX-I medium supplemented with serum (10%) and Insulin (10 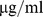). For infection 0.5 million Mm5MT cells were plated in a T25 flask in the presence of 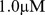 Hydrocortisone. Next day the cells were treated with 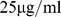 of Mitomycin C in serum-free medium for two hours. Then 0.5 million NMuMG cells were cultured on top of the Mitomycin C treated Mm5MT cells in the presence of 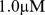 Hydrocortisone. Three days later the mixed cell culture was treated with 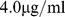 of Puromycin to remove Mm5MT cells. The remaining cells (NMuMG) were grown till passage 8 before the isolation of genomic DNA for integration site mapping (Figure S9). By using a primer pair, which was specific to MMTV in Mm5MT and did not amplify endogenous MMTV sequences in NMuMG cells, it was confirmed that NMuMG cells got infected. The integration sites were measured by two methods: either shearing the DNA with sonication and blunt end ligation of adapters as described previously [31] or cutting the DNA with Nla-III and ligation of adapters with sticky ends. 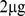 of genomic DNA was digested with 20 units of Nla-III (New England Biolabs) at 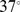 C for overnight in a 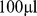 reaction. 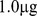 of purified digested DNA was ligated with 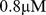 of splinkerette adapter using 10 units of T4 DNA ligase (Roche Applied Science) in a 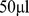 reaction. The splinkerette adapter was prepared by annealing equimolar amounts (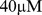 each) of Universal LS (CCTATAGTGAGTCGTATTATAATTTTTTTTTCAAAAAAA) and Nla-III US (GTTCCCATGGTACTACTCATATAATACGACTCACTATAGGCATG) oligos. The ligated DNA was cut with Dra-I (New England Biolabs). The ligation reactions were amplified in two rounds of PCR to generate high throughput sequencing libraries (for details see Table S7). Sequencing was done on an Illumina HiSeq 2000 instrument to obtain single 100 bp reads. The reads contained ends of MMTV LTR and the neighboring genomic DNA. The genomic DNA sequences were extracted from sequencing reads, and aligned against mouse genome assembly mm9 using Bowtie [59] to determine the sites and orientation of integrations.

### Data preprocessing

#### Matched random controls

Given an integration dataset (either one of SB, PB, or MMTV), each integration in that dataset was matched to 10 random controls. These random controls were subject to a number of criteria. First, specifically for SB and PB, matched controls were restricted to loci containing the system-specific integration motif (TA and TTAA respectively). Second, the distance of the matched control to the nearest restriction site upstream of the integration was required to be the same as that of the integration itself. Third, matched controls were not allowed to fall within ‘unmappable’ regions. Here, unmappable regions were defined in a dataset-dependent manner. Given an integration dataset (either one of SB, PB, or MMTV), the sequence read length 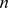 was determined. Then, the mouse genome (mm9) was cut up into all possible sequences of length 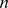. These artificial reads were mapped to the mm9 genome using the same tool and parameter settings as used to generate the integration datasets (see above). Unmappable regions were then defined as regions that did not have any reads mapped to them using this approach, and controls were excluded from these regions.

#### ChIP-seq

To maximize the comparability of the ChIP-seq datasets, they were processed from the sequence read archives as obtained from GEO [60], where possible, in exactly the same way. Sequence read archives were converted to FASTA format and then aligned against mouse genome assembly mm9 using Bowtie 0.12.7 [59], allowing at most 2 mismatches in end-to-end alignment (the following settings were used: -M 1 best tryhard -v 2 chunkmbs 1024). Duplicate reads were removed, and a 25 bp coverage was computed by counting the number of reads in 25 bp consecutive bins. These coverage profiles were normalized to a sequencing depth of 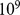, smoothed (running mean with window n = 6), and sampled (to 100 bp spacing). Then, all available input DNA datasets (9 in total) were collected and clustered based on the coverage profile. A cluster of 6 input DNA datasets with a correlation of at least 0.97 was selected to be pooled and used as control for all non-histone mark features. This was done because 1) not for all datasets controls were available, 2) not for all datasets controls of the same type (input DNA, GFP, mock IP, etc.) were available. The 6 selected input DNA datasets were used as a control dataset. First, after normalizing to total read count, they were averaged to obtain a pooled control coverage profile, and then used to normalize all non-histone mark features by computing log2(signal/control). For the histone marks, a similar approach was taken, using all available pan-H3 datasets (2 in total).

#### Bisulfite sequencing

Bisulfite sequencing reads [61] were processed using Methylcoder [62], with “mismatches = 0”, and Bowtie to align the reads, with “-M 1 best tryhard -v 2 chunkmbs 1024”. A coverage profile was computed by selecting only methylation context ‘CG’ for CpG methylation, and counting in 25 bp consecutive bins the numbers of unconverted Cs 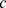, converted Cs 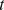, and methylable basepairs 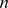, and calculating 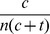. The resulting profile was smoothed and sampled as explained above.

#### RNA-seq

The RNA-seq reads were processed using Cufflinks [63] to compute the log2(FPKM+1) for each gene, where FPKM refers to the number of fragments per kilobase of exon per million fragments mapped.

#### Preprocessing of microarray datasets

The H3K9me2, late replication timing data, and LaminB1 data were downloaded from GEO and processed as in the corresponding publications [64]–[66].

### Data analysis

#### Gene alignments (Figure 1A)

Gene locations were retrieved from the Ensembl database (release 66). Partially overlapping genes were removed (40%). In case of complete overlap, the larger gene was retained. The remaining genes (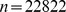) were aligned with respect to transcription start sites and transcription termination sites. For each integration dataset, integrations and controls (see above) were counted in equal-sized bins outside genes, and gene length dependent bins within genes. Then, for each bin a ratio was computed of integration counts versus control counts. This ratio was normalized by multiplication with the ratio of control dataset size and integration dataset size. Then the base 2 logarithm was taken.

#### Bias with respect to genes and transcripts (Figure 1B)

For genes (Ensembl release 66), integrations and controls (see above) were counted within genic regions, TSS upstream regions (defined as the union of those regions within 1 kb upstream of a TSS), TSS downstream regions (defined as the union of those regions within 1 kb downstream of a TSS), and everything else. Note that these classes can overlap. Then, the ratio was computed of integration counts versus control counts. This ratio was normalized by multiplication with the ratio of control dataset size and integration dataset size. Then the base 2 logarithm was taken. A similar approach was taken for the transcript-related classes 5′UTR, 3′UTR, exon, and intron.

#### Bias with respect to gene expression (Figure 2)

Genes were divided into five quantiles, based on their expression level. For SB and PB, Group 1 consisted of all genes with an FPKM of zero in the RNA-Seq dataset. The remaining genes were divided across four equal quantiles. The NMuMG microarray expression dataset for MMTV was divided according to the same expression quantiles. Subsequently, the same approach as above was taken for determining the numbers of integrations within each of these subsets of genes.

#### Topologically associated domains (Figure 3)

Domain definitions were adopted from [39]. Interfaces between TADs and TAD boundaries were aligned, and integrations and controls (see above) were counted until halfway into the TAD as well as halfway into the TAD boundary region. Then, a log2 ratio between the two was calculated.

#### Association of integration occurrence with genome-wide features (Figure 4)

For each feature and integration, a feature score was computed at exponentially increasing distances (scales) from that integration. For all deep sequencing based features, this was done by taking the mean normalized read count within a 200 bp window, from the genome-wide binned read count profiles computed as outlined above. For CpG islands, genes, and TSSs, this score was calculated by taking the negative log2 transformed distance (+1) to the nearest CpG island, gene, or TSS. For the microarray features, this score represented the value of the nearest probe. The Hi-C score was computed as follows. Normalized Hi-C contact frequency matrices (20 kb bins) were downloaded from (http://chromosome.sdsc.edu/mouse/hi-c/mESC.norm.tar.gz). For each locus, defined by an integration and a scale, average contact frequencies as a function of distance from that locus, were calculated within a window of 400 kb. The Hi-C 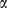 was computed as the slope of a linear regression fit to the log10 transformed distances and log10 transformed contact frequencies. Once the feature scores for all triples (feature, scale, and integration) were calculated, feature scores were rank-normalized on a per-feature basis, and a *t*-score, 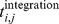, was computed for each scale 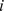 and feature 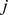 as follows:
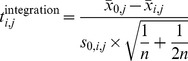
(1)where
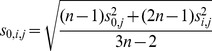
(2)


Here, 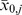 represents the mean of all scores of feature 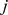 at the sites of integration, 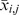 the mean of all scores of feature 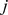 at scale 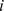, and 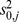 and 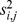 their respective variances. 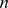 represents the number of integrations.

The same was done for the 10 sets of control loci, since we have 10 matched controls for each integration. This resulted in 1 set of integration *t*-scores, 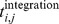, and 10 sets of control *t*-scores, 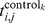. Then, the difference between the set of integration *t*-scores and the mean of the control *t*-scores was computed (and plotted in Figure 4):
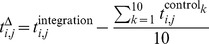
(3)


To compute *p*-values for the 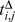, we can take advantage of the fact that for large degrees of freedom, the 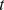-distribution converges to the standard normal distribution. Thus, the calculated *t*-scores can be interpreted as normally distributed with mean 0 and standard deviation 1, i.e. as 1 set of integration 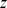-scores, 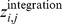, and 10 sets of control 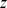-scores, 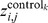:
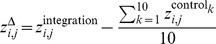
(4)


Now, note that if:
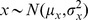


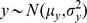


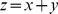



Then
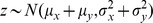



Note furthermore, that if 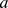 is some constant and
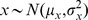


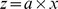



Then
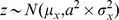



Therefore
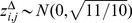
(5)


The *p*-values for this distribution are readily computed.

#### Conditional independencies (Figure 5)

For each integration, the feature values were normalized by division with the average of the 10 corresponding control feature values (or subtraction in case of log-transformed feature values). Then, all data was discretized into three quantiles. To identify conditional independencies in the discretized data we took the approach of Bayesian networks [41]. Bayesian networks specify for each feature a set of other features, the Markov blanket. A Markov blanket of a feature represents a minimal set of features sufficient to characterize the distribution of that feature. More formally, given its Markov blanket, a feature is conditionally independent from all other features. In addition to modeling conditional independencies, Bayesian networks can capture nonlinear effects, such as the changes of sign of association across scales in Figure 4. For inferring Bayesian networks, we used BANJO [40]. Since BANJO relies on stochastic optimization (simulated annealing), we inferred Bayesian networks for 400 bootstraps of size 20000. For each bootstrap, the Markov blanket of the integration proximity node was determined, and the importance of a feature was represented in two dimensions: First, the fraction of bootstraps a feature occurred in a resulting Markov blanket. This conveys the degree of confidence we have that this feature is truly relevant. Second, the conditional mutual information between integration proximity and a feature from its Markov blanket, given the other features in its Markov blanket, averaged across all inferred Markov blankets. This coveys the strength of association between integration proximity and this feature, where this strength could not be explained by any of the other features that were inferred to be relevant.

#### Unselected vs. CIS integrations (Figure 6A)

For each of the three systems, CISs were called on the tumor screens, using the approach outlined in [33], with a 30 kb kernel width and a 5% Bonferroni-corrected *p*-value threshold. CIS regions were defined as those regions where the Gaussian smoothing kernel exceeded the significance threshold, extended on either side with 30 kb (kernel width used for calling the CISs). CIS integrations were defined as those integrations from the tumor screen that fell within a CIS region, and log2 ratios of unselected integrations and CIS integrations were calculated as described above, replacing control loci with CIS integrations.

#### Activating/repressing CISs (Figure 6B)

CISs were called as outlined above. A CIS was defined to be an activating CIS if its peak location was either not within a gene, or within a gene and orientation-wise homogeneous, requiring 90% of integrations falling within a CIS to be of the same orientation.

#### CIS region bias of unselected integrations (Figure 6C)

For the union of genic regions (+/−100 kb) it was counted how many unselected integrations were found within CIS regions, how many outside CIS regions, and the corresponding numbers of expected unselected integrations in those regions; *p*-values were calculated based on a binomial test. This procedure outlined for genic regions was repeated for the whole genome, and for intergenic regions.

#### Potentially spurious CISs (Figure 6D)

For all CISs, it was determined how many CIS integrations and unselected integrations fell within the corresponding CIS region. CIS regions were defined as above. To determine whether a CIS contained significantly more CIS integrations than unselected integrations, a one-sided binomial test was performed, testing the significance of 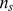 successes in 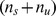 trials, and corrected for multiple testing (FDR). Here, 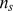 is the number of tumor screen integrations within a CIS region, and 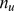 is the number of unselected integrations within a CIS region. Low (high) *p*-values correspond to true (spurious) CISs. The probability of success for each binomial test was defined as 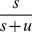, where 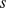 is the tumor screen dataset size, and 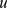 is the unselected integration dataset size.

CISs were annotated with the name of the gene of nearest TSS, where the TSSs were restricted to Ensembl IDs that had corresponding UCSC, EntrezGene, MGI, and UniGene IDs. To determine whether the non-significant CISs tended to be farther away from genes, the genome-wide TSS density was estimated using a Gaussian smoothing kernel (standard deviation 1 Mb), and sampled at 1 kb intervals. Integrations were then mapped to the nearest sampled density estimation point, and a Mann-Whitney U test was performed on selected integrations within CIS regions versus unselected integrations within CIS regions.

## Supporting Information

Figure S1 Distribution of non-canonical recognition sequences for PB and SB. The non-canonical recognition sequences are generally relatively similar to the primary motifs and often differ by only one nucleotide, such as CTAA, TTAG, TAAA for PB and TG, CA, and AA for SB.
(EPS)

Figure S2 Sequence logos for all integration datasets, and de novo motif search near integration sites. For a dataset, all integrations were aligned with respect to locus and orientation, and sequence logos were derived for the set of sequences spanning a distance from 15 bp upstream of integrations to 15 bp downstream of integrations. As expected, PB and SB strongly prefer TTAA and TA respectively. This preference is however not exclusive since these motifs account for 93% (PB; TTAA) and 94% (SB; TA) of the total number of integrations. For SB, in addition to the recognition sequence TA centered around the integrations, a preference for A and T exactly 4 bp upstream and 4 bp downstream can be observed. MMTV is the least sequence specific of the three systems. Also, below each sequence logo are shown the top 10 motifs found within 50 bp upstream and downstream of the integration, as determined using the HOMER software for motif discovery [34] are shown.
(EPS)

Figure S3 Gene alignment plots for all systems. For all systems, the blue and red lines refer to unselected integrations. The black lines refer to singleton integrations for all profiles, the black dotted lines to CIS integrations. Note that no unselected MuLV data was available. Therefore, the set of singleton MuLV integrations was used as a proxy for an unselected integration profile.
(EPS)

Figure S4Distribution of integrations across genes and transcripts. Distribution of A) unselected integrations, and B) CIS integrations across genes and transcripts. For genes, we distinguish between integrations within genes (genic), within 2 kb upstream of the TSS (TSS_upstream), within 2 kb downstream of the TSS (TSS_downstream), and other integrations (other). For transcripts, we distinguish between integrations in 5′UTR, 3′UTR, exons and introns. The color scale blue-gray-red represents increasing numbers of integrations, relative to expected (left), or the integration orientation bias relative to genes, from antisense to sense (right). Associations that are not significant (binomial test; FDR-corrected 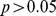) are white. Note that orientation bias of unselected integrations is present but relatively weak, compared to the CIS integrations (numeric differences in color scale annotation).(EPS)

Figure S5Orientation bias of integrations with respect to various genome-wide features. Orientation bias with respect to various (epi)genomic features for A) all integrations, B) integrations in sense orientation relative to the nearest TSS, and C) integrations in antisense orientation relative to the nearest TSS. Measure of association is a normalized *t*-score (see Material and Methods), computed on rank-normalized feature values, visualized on a blue-gray-red scale from negative to positive *t*-scores. A positive (negative) *t*-score for a certain feature 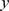 means that for that particular feature the mean values in a 200 bp window upstream of the integrations are on average higher (lower) than the mean values in a 200 bp window downstream of the integrations.(EPS)

Figure S6 Overall orientation bias of integrations with respect to (epi)genomic features. For each heatmap and system in the previous figure, the fraction of positive feature *t*-scores is calculated, and 0.5 is subtracted. The resulting score represents the overall orientation bias of the system with respect to the features, positive (negative) values implying inserting generally oriented away (toward) regions of high signal. Reported *p*-values are based on two-sided binomial tests and FDR-corrected. This shows that the general preference for inserting oriented towards regions of high signal can not be explained by an orientation bias of the integrations relative to genes in the case of SB, and only partly in the case of PB.
(EPS)

Figure S7MMTV macrofeatures. Association of the unselected MMTV integrations with a limited set of macrofeatures across different scales, showing that this set of features also behave as macrofeatures not only in SB and PB, but also in MMTV, an unrelated species. Measure of association is the *t*-score, computed on rank-normalized feature values, visualized on a blue-gray-red scale from negative to positive *t*-scores. Associations that are not significant (FDR-corrected 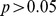) are white. *t*-scores are transformed with a hyperbolic arcsine function for better visualization. A positive (negative) *t*-score for a certain scale 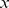 and feature 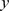 means that for that particular feature the mean values in a 200 bp window around the integrations are on average higher (lower) than the mean values in a 200 bp window around the points at a distance of 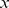 upstream and downstream from the integration.(EPS)

Figure S8Comparing SB and PB integration profiles to epigenetic features in mEFs and mESCs. mEF data was obtained from [48]–[52]. For both the mEF and mESC features, *t*-scores were computed for feature scores at integration loci vs. feature scores at control loci, and plotted in the 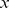-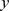 plane. Feature scores at integration and control loci were computed as described in the Material and Methods section. This shows that associations are generally highly similar for mEFs and mESCs, i.e. there is a strong correlation of *t*-scores in the 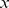-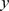 plane, suggesting that cell type specificities are weak at best. It is noteworthy that although this correlation is strong, associations for mEFs are generally somewhat weaker than the associations for mESCs, indicating that weak cell type specificities do exist.(EPS)

Figure S9Experimental design. Generation of unselected integration datasets for transposons and MMTV. A) A construct with IRs for both SB and PB and the coding sequence for eGFP driven by CMV promoter was co-transfected in mouse ES cells with either SB or PB transposase. After 48 hr of transfection, 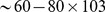 cells were sub-cultured for 18 days (20 days in total after transfection) before the isolation of DNA for integration site analysis using splinkerette PCR. B) Mitomycin C treated Mm5MT cells (which produce MMTV virus) were co-cultured with Puromycin resistant NMuMG cells for 72 hr. After that, cells were passaged and treated with Puromycin to remove mitotically inactive Mm5MT cells. The infected NMuMG cells were further propagated for three weeks and DNA was isolated for integration site mapping.(EPS)

Table S1 List of statistical procedures used in the main text.
(XLS)

Table S2 FDR-corrected *p*-values associated with statistical tests performed for Figures 1B, 1C, 6A, 6C.
(XLS)

Table S3 Macrofeatures and microfeatures.
(XLS)

Table S4SB true and spurious CISs. Column name ‘nU’ refers to the number of unselected integrations within the CIS region on chromosome ‘chr’, starting at ‘start’ and ending at ‘end’. ‘nC’ refers to the corresponding number of CIS integrations. The *p*-values in ‘pvalue’ were calculated by a one-sided binomial test of ‘nC’ successes in ‘nC’+‘nU’ trials, and corrected for multiple testing (FDR). Low (high) *p*-values correspond to true (spurious) CISs. The probability of success for each binomial test was defined as 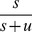, where 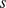 is the tumor screen dataset size, and 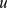 is the unselected integration dataset size. Each CIS region is mapped to its nearest TSS (columns ‘symbol’ and ‘ensemblID’).(XLS)

Table S5PB true and spurious CISs. Column name ‘nU’ refers to the number of unselected integrations within the CIS region on chromosome ‘chr’, starting at ‘start’ and ending at ‘end’. ‘nC’ refers to the corresponding number of CIS integrations. The *p*-values in ‘pvalue’ were calculated by a one-sided binomial test of ‘nC’ successes in ‘nC’+‘nU’ trials, and corrected for multiple testing (FDR). Low (high) *p*-values correspond to true (spurious) CISs. The probability of success for each binomial test was defined as 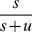, where 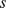 is the tumor screen dataset size, and 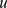 is the unselected integration dataset size. Each CIS region is mapped to its nearest TSS (columns ‘symbol’ and ‘ensemblID’).(XLS)

Table S6MMTV true and spurious CISs. Column name ‘nU’ refers to the number of unselected integrations within the CIS region on chromosome ‘chr’, starting at ‘start’ and ending at ‘end’. ‘nC’ refers to the corresponding number of CIS integrations. The *p*-values in ‘pvalue’ were calculated by a one-sided binomial test of ‘nC’ successes in ‘nC’+‘nU’ trials, and corrected for multiple testing (FDR). Low (high) *p*-values correspond to true (spurious) CISs. The probability of success for each binomial test was defined as 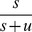, where 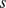 is the tumor screen dataset size, and 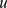 is the unselected integration dataset size. Each CIS region is mapped to its nearest TSS (columns ‘symbol’ and ‘ensemblID’).(XLS)

Table S7Amplification of samples for mapping integrations by high throughput Illumina sequencing. For PCR-1 Thermo-Start Taq DNA polymerase (ThermoScientific, cat # AB0908F) was used whereas for all other PCR reactions Phusion High-Fidelity DNA Polymerase (ThermoScientific, cat # F-534L) was used. 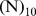 represents the index sequences used to tag individual samples for high throughput Illumina sequencing.(DOC)
