# Supplementary material for: Chromatin Landscapes of Retroviral and Transposon Integration Profiles
Source: PLoS Genet. 2014 Apr 10;10(4):e1004250. doi: 10.1371/journal.pgen.1004250 (PMC3983033; doi:10.1371/journal.pgen.1004250)
Supplement: Table S7 — Amplification of samples for mapping integrations by high throughput Illumina sequencing. For PCR-1 Thermo-Start Taq DNA polymerase (ThermoScientific, cat # AB0908F) was used whereas for all other PCR reactions Phusion High-Fidelity DNA Polymerase (ThermoScientific, cat # F-534L) was used. represents the index sequences used to tag individual samples for high throughput Illumina sequencing. (DOC) [file pgen.1004250.s016.doc]

| **PCR** | **Template** | **Primers (5’->3’)** | **Number of cycles (annealing temp.)** |
| --- | --- | --- | --- |
| *Mapping of PB integrations (4 x for each technical replicate)* | | | |
| PCR-1 | 3 µl of the ligation mix | Forward primer P-7 (GTTCCCATGGTACTACTCATA) and  PB-outer-F-2 (TTTTACGCATGATTATCTTTAACGTACGTC) | 10 (67°C)  and  15 (65°C) |
| PCR-2 | 1 μl of PCR-1 | S1-algemeen-1-BC- (ACACTCTTTCCCTACACGACGCTCTTCCGATCT(N)_10_CGTCACAATATGATTATCTTTCTAGG) and  Solexa-R-T7 primer (CAAGCAGAAGACGGCATACGAGATCTACTCATATAATACGACTCACTATAGG) | 10 (60°C) |
| PCR-3 | 1 μl of PCR-2 | Solexa-Adaptor-SE1 (AATGATACGGCGACCACCGAGATCTACACTCTTTCCCTACACGACGCTCTTCCGATCT) and  Solexa-R-T7 primer (CAAGCAGAAGACGGCATACGAGATCTACTCATATAATACGACTCACTATAGG) | 12 (60°C) |
| *Mapping of SB integrations (4 x for each technical replicate)* | | | |
| PCR-1 | 3 µl of the ligation mix | Forward primer P-7 (GTTCCCATGGTACTACTCATA) and  SB-L1 (CTGGAATTTTCCAAGCTGTTTAAAGGCACAGTCAAC) | 10 (67°C)  and  15 (65°C) |
| PCR-2 | 1 μl of PCR-1 | Solexa-F-SB-BC- (AATGATACGGCGACCACCGAGATCTACACTCTTTCCCTACACGACGCTCTTCCGATCT(N)_10_GTGTATGTAAACTTCCGACTTCAAC) and  Solexa-R-T7 primer (CAAGCAGAAGACGGCATACGAGATCTACTCATATAATACGACTCACTATAGG) | 20 (60°C) |
| *Mapping of MMTV integrations (4 x for each technical replicate)* | | | |
| PCR-1 | 3 µl of the ligation mix | Forward primer P-7 (GTTCCCATGGTACTACTCATA) and  Reverse primer MMTV 4 (CCTTGGCTGCTTCTCCCCTAGGT) | 10 (67°C)  and  15 (65°C) |
| PCR-2 | 1 μl of PCR-1 | Solexa-F-MMTV-BC- (AATGATACGGCGACCACCGAGATCTACACTCTTTCCCTACACGACGCTCTTCCGATCTAT(N)_10_ACCATTTCTGCTGCAGGC) and  Solexa-R-T7 primer (CAAGCAGAAGACGGCATACGAGATCTACTCATATAATACGACTCACTATAGG) | 20 (60°C) |
